# Supplementary material for: Observationally quantified reconnection providing a viable mechanism for active region coronal heating
Source: Nat Commun. 2018 Feb 15;9:692. doi: 10.1038/s41467-018-03056-8 (PMC5814417; doi:10.1038/s41467-018-03056-8)
Supplement: Supplementary file 1 — Supplementary Information [file 41467_2018_3056_MOESM1_ESM.pdf]

Observationally Quantified Reconnection Providing a Viable Mechanism for Active Region Coronal Heating

Kai E. Yang[1,2,3], Dana W. Longcope[2], M. D. Ding[1,3], and Yang Guo[1,3]

[1]School of Astronomy and Space Science, Nanjing University, Nanjing 210023, China
[2]Physics Department, Montana State University, Bozeman, MT 59717, USA
[3]Key Laboratory for Modern Astronomy and Astrophysics (Nanjing University), Ministry of Education, Nanjing 210023, China

Supplementary Figures

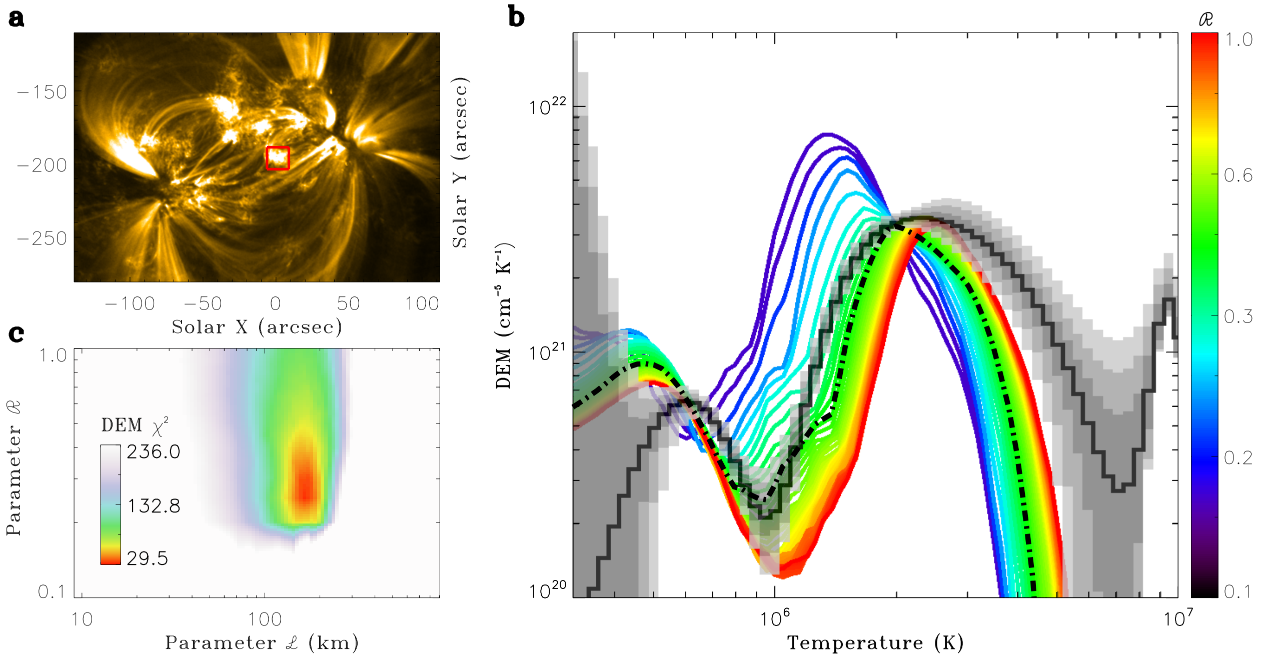

Supplementary Figure 1 | DEM distribution from the model and the discrepancy χ^2 between the model and observations. **a**, The image at EUV 171 Å observed by AIA. The red box indicates the area where the DEM is calculated and shown in **b**. **b**, The DEM distribution calculated from the model by fixing $\mathscr{L} = 160$ km but varying the ratio of heat scale length, $\mathscr{R}$ (see the color bar). The solid black curve is for the DEM inferred from the AIA observations at six wavelengths averaged over 12 minutes. The gray areas with different transparencies represent the results with 1σ, 2σ and 3σ from the Monte-Carlo test. The dash-dotted line is the curve from the model with $\mathscr{R} = 0.3$. **c**, The discrepancy χ^2 between the modelled DEM and the observed one plotted as a color scale over parameter space.

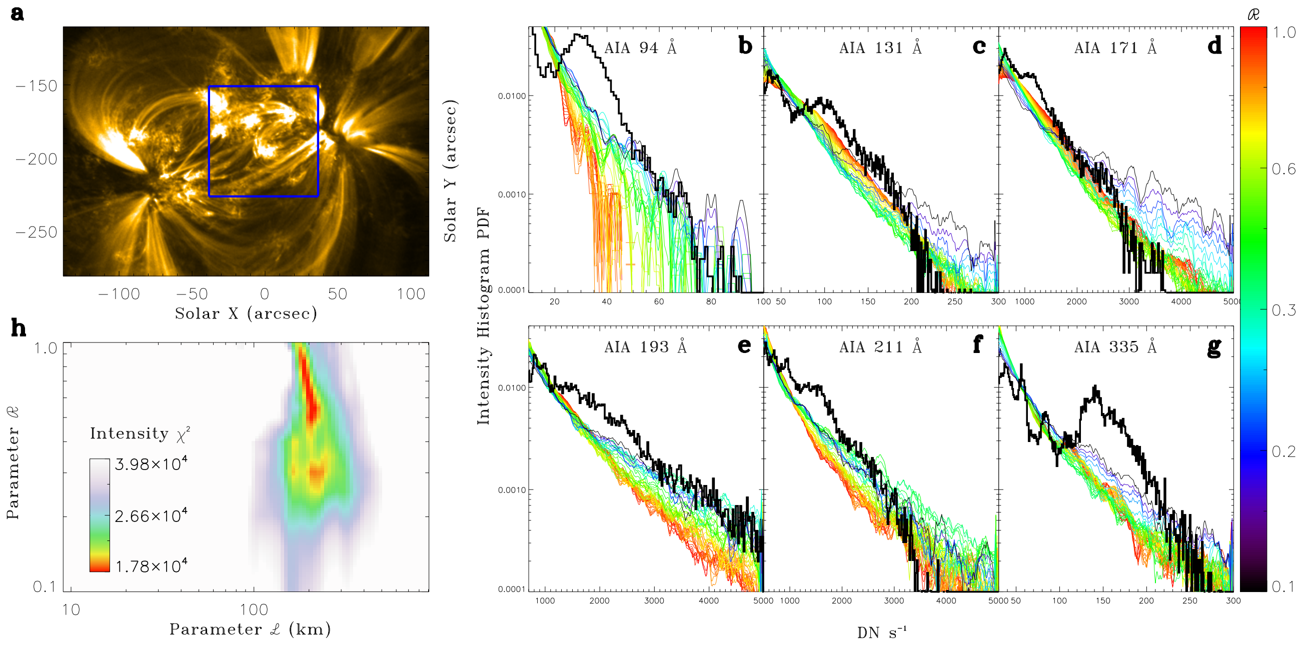

Supplementary Figure 2 | Intensity histogram from the model and the discrepancy χ^2 between the model and observations. a, The image at EUV 171 Å observed by AIA. The blue box refers to the area over which the discrepancy is computed between the intensity histograms from the model and observations as shown in **b–g**. **b–g**, The intensity histogram calculated from the model by fixing $\mathscr{L} = 160$ km but varying the ratio of heat scale length, $\mathscr{R}$ (see the color bar). The solid black curve stands for the intensity histogram from the AIA observations at six wavelengths averaged over 12 minutes. **h**, The sum of the Pearson's χ^2 of the intensity histogram between model and observations over all of the six wavelengths, $\chi^2 = \sum_i \chi_i^2$, where i is the index of the six AIA channels. It is plotted as a color scale over parameter space.
